# Gated auditory speech perception: effects of listening conditions and cognitive capacity

**DOI:** 10.3389/fpsyg.2014.00531

**Published:** 2014-06-02

**Authors:** Shahram Moradi, Björn Lidestam, Amin Saremi, Jerker Rönnberg

**Affiliations:** ^1^Linnaeus Centre HEAD, The Swedish Institute for Disability Research, Department of Behavioral Sciences and Learning, Linköping UniversityLinköping, Sweden; ^2^Department of Behavioral Sciences and Learning, Linköping UniversityLinköping, Sweden; ^3^Division of Technical Audiology, Department of Clinical and Experimental Medicine, Linköping UniversityLinköping, Sweden; ^4^Cluster of Excellence “Hearing4all”, Department for Neuroscience, Computational Neuroscience Group, Carl von Ossietzky University of OldenburgOldenburg, Germany

**Keywords:** gating paradigm, auditory perception, consonant, word, final word in sentences, silence, noise

## Abstract

This study aimed to measure the initial portion of signal required for the correct identification of auditory speech stimuli (or isolation points, IPs) in silence and noise, and to investigate the relationships between auditory and cognitive functions in silence and noise. Twenty-one university students were presented with auditory stimuli in a gating paradigm for the identification of consonants, words, and final words in highly predictable and low predictable sentences. The Hearing in Noise Test (HINT), the reading span test, and the Paced Auditory Serial Attention Test were also administered to measure speech-in-noise ability, working memory and attentional capacities of the participants, respectively. The results showed that noise delayed the identification of consonants, words, and final words in highly predictable and low predictable sentences. HINT performance correlated with working memory and attentional capacities. In the noise condition, there were correlations between HINT performance, cognitive task performance, and the IPs of consonants and words. In the silent condition, there were no correlations between auditory and cognitive tasks. In conclusion, a combination of hearing-in-noise ability, working memory capacity, and attention capacity is needed for the early identification of consonants and words in noise.

## Introduction

Previous studies have attempted to establish isolation points (IPs), that is, the initial portion of a specific acoustic signal required for the correct identification of that signal, in silent conditions (see Grosjean, 1980). An *IP* refers to a given point in the total duration of a speech signal (i.e., a word) that listeners are able to correctly guess the identity of that signal with no change in their decision after hearing the reminder of that signal after that given point. In the present study, we investigated the IPs of different types of spoken stimuli (consonants, words, and final words in sentences) in both silence and noise conditions, in order to estimate the extent to which noise delays identification. In addition, a cognitive hearing science perspective was used to evaluate the relationships between explicit cognitive variables (working memory and attentional capacities), speech-in-noise perceptual ability, and IPs of spoken stimuli in both silence and noise.

## The initial portion of stimuli required for correct identification of consonants, words, and final words in sentences

### Consonant identification

The specific combined features of place (the place in the vocal tract that an obstruction occurs), manner (the configuration of articulators, i.e., tongue or lips, when producing a sound), and voicing (absence or presence of vocal fold vibration) constitute a given consonant. Listeners can correctly identify a consonant when these particular features are available (Sawusch, 1977). Smits (2000) reported that the location and spread of features for stops, fricatives, and nasals are highly variable. In a French gating-paradigm study, Troille et al. (2007) showed that for a 120-ms /z/ consonant, identification occurred about 92 ms before its end.

Noise in combination with the acoustic features of consonants may cause a perceptual change, such that the noise may be morphed together with the consonant, masking or adding consonant features, thereby changing the percept into another consonant (Miller and Nicely, 1955; Wang and Bilger, 1973; Phatak and Allen, 2007). As a result, the number of correctly identified consonants in noise is reduced (Wang and Bilger, 1973; Phatak and Allen, 2007). Phatak and Allen (2007) reported that consonant identification in white noise falls into three categories: a set of consonants that are easily confused with each other (e.g., /f v b m/), a set of consonants that are intermittently confused with each other (e.g., /n p g k d/), and a set of consonants that are hardly ever confused with each other (e.g., /t s z /). Based on the results of Phatak and Allen (2007) showing that noise impacts differently on different consonants, one may predict that the influence of noise should be larger for the consonants that are more easily confused with each other. Furthermore, the signal-to-noise ratio (SNR) required for the identification of consonants varies across consonants (Miller and Nicely, 1955; Woods et al., 2010). We therefore expect that, compared with silence, noise will generally delay the correct identification of consonants.

### Identification of isolated words

Word identification requires an association between an acoustic signal and a lexical item in long-term memory (Lively et al., 1994). According to the cohort model (Marslen-Wilson, 1987), initial parts of a speech signal activate several words in the lexicon. As successively more of the acoustic signal is perceived, words in the lexicon are successively eliminated. Word identification occurs when only one word candidate is left to match the acoustic signal. Gating paradigm studies have generally demonstrated that word identification occurs after a little more than half of the duration of the whole word (Grosjean, 1980; Salasoo and Pisoni, 1985).

Identification of isolated words is poorer in noise than in silence (Chermak and Dengerink, 1981; Dubno et al., 2005). As the main constituents of words, some vowels (Cutler et al., 2005) and consonants (Woods et al., 2010) are highly affected by noise. For instance, Parikh and Loizou (2005) showed that whereas /o/ had the lowest identification score in a noisy condition compared to other vowels, /i/ had the highest identification score. Presentation of /o/ in a noisy condition activated perception of other vowels like /U/. Based on the findings of Parikh and Loizou (2005), noise has differential effects on identification of different vowels (similar to consonants), meaning that the combination of vowels and consonants with noise activates other vowels and consonants, which disturbs the mapping of the input signal with the representations in the mental lexicon. We expect that the addition of these noise-induced extra-activated candidates will delay IPs, as more acoustic information will be needed to map the signal with the phonological representations in the mental lexicon. In addition, noise is likely to be detrimental to the success of this mapping, as it results in a lower intelligibility.

### Identification of final words in sentences

When words are presented in sentences, listeners can benefit from the syntactic structure (Miller and Isard, 1963) and semantic context in congruent sentences (Kalikow et al., 1977), which in turn can speed up target word identification in comparison with word-alone presentation (Miller et al., 1951; Grosjean, 1980; Salasoo and Pisoni, 1985). This improvement in word identification occurs because contextual factors inhibit the activation of other lexical candidates that are a poorer fit for the linguistic context (Marslen-Wilson, 1987).

The predictability of sentences is a key variable for final word identification in sentences. The estimation of word predictability is derived from a “cloze task procedure” (Taylor, 1953) when subjects are asked to perform a sentence completion task with the final word is missing. For instance, the word “bird” in the sentence “a pigeon is a kind of bird” is an example of a highly predictable word but in the sentence “she pointed at the bird” it is as an example of a low predictable word. It should be noted that the highly predictable and low predictable words differ from anomalous words, wherein words are randomly substituted. Regarding the example above, the word “bird” is incongruous in the sentence “The book is a bird.” Final words are easier to identify in meaningful sentences than in semantically anomalous sentences (Miller and Isard, 1963). Highly predictable sentence contexts enhance one's capability to disambiguate final words compared with low predictable sentence contexts (Kalikow et al., 1977).

Prior context facilitates word identification in noise (e.g., Grant and Seitz, 2000); when highly predictable sentences are heard, the auditory thresholds for word identification are lowered (Sheldon et al., 2008; Benichov et al., 2012). Final word identification in noise is different from tests on sentence comprehension in noise (e.g., the Hearing in Noise Test [HINT], Nilsson et al., 1994; Hällgren et al., 2006). The latter requires the listener to repeat the entirety of sentences, in an adaptive procedure. However, final word identification tasks are usually presented at a constant SNR, and require participants to predict which word will come at the end of the sentence, and therefore demands less cognitive effort. They thus differ in the retrieval demands they put on explicit resources such as working memory (Rönnberg et al., 2013).

### Cognitive demands of speech perception in silence and noise

According to the Ease of Language Understanding (ELU) model (Rönnberg et al., 2008), working memory acts as an interface between incoming signals and the mental lexicon. Working memory enables the storage and processing of information during online language understanding. In this model, the incoming signal automatically feeds forward at a sub-lexical (syllable) level in rapid succession to match the corresponding phonological representation in the mental lexicon (cf. Poeppel et al., 2008; Rönnberg et al., 2013). This process of syllabic matching is assumed to demand less working memory capacity for normal-hearing people under optimum listening conditions, resulting in rapid and implicit online language processing. However, if the incoming signal is poorly specified or distorted (e.g., in noisy conditions), a mismatch (or non-match, cf. Rönnberg et al., 2013 for a detailed discussion on the match/mismatch issue) will occur with the phonological representation in the mental lexicon. The rapid and implicit process of lexical access is temporarily disturbed under such conditions. In such cases, explicit and deliberate cognitive processes (i.e., inference-making and attentional processing) are invoked to compensate for this mismatch in order to detect or reconstruct the degraded auditory signal. Previous studies have shown that attentional and inference-making processes greatly depend on working memory capacity (Kane and Engle, 2000; De Neys et al., 2003). Independent support for the ELU model (Rönnberg et al., 2008) comes from studies showing two auditory cortical mechanisms of processing: an automatic segregation of sounds, and an attention-demanding network that analyzes the acoustic features of incoming auditory signals (Petkov et al., 2004; Snyder et al., 2006, see also Rönnberg et al., 2013). Röer et al. (2011) reported that auditory distraction disturbs the automatic connection of auditory stimuli to the phonological representations in long-term memory.

Previous research has supported the notion that working memory capacity is crucial for speech perception in adverse listening conditions (for recent reviews, see Rönnberg et al., 2010, 2013; Mattys et al., 2012). Unfavorable listening conditions place higher demands on working memory processing (Lunner et al., 2009), and less resources are therefore available for the storage of incoming signals (Rabbitt, 1968).

Attentional capacity of listeners is also a cognitive function that plays a critical role in speech perception under degraded listening conditions (Carlyon et al., 2001; Shinn-Cunningham and Best, 2008; Mesgarani and Chang, 2012). In degraded listening conditions, attention is focused on the signal's frequency (Dai et al., 1991), the spatial spectrum (Mondor et al., 1998; Boehnke and Phillips, 1999), one channel of information (Conway et al., 2001), or the switching between channels of information (Colflesh and Conway, 2007). This focus of attention enables the segregation of different types of auditory competitors for speech understanding and subsequent memory encoding (cf. Rönnberg et al., 2008, 2013; Sörqvist and Rönnberg, 2012; Sörqvist et al., 2012).

### The present study

The general purpose was to study how large the initial portion of the stimulus needs to be in order for correct identification, and therefore how demanding the perception is, as an effect of how easy the signal is to discriminate and predict. IPs refer to how large the initial portion of the entire signal that is needed for correct identification. Hence, IPs specify how much of the entire signal is required for correct identification, and thereby how quickly the stimuli are identified. It can be assumed that the identification of stimuli is less demanding if the stimuli are identified earlier. Therefore, IPs should allow us to estimate the amount of cognitive demand needed for correct identification of speech stimuli in silence versus in noise, which lowers discriminability, and under different levels of predictability (e.g., due to lexical and sentential context). In turn, this should be reflected in correlations with measures of explicit cognitive functions.

The general purpose encompasses two aims. The first aim was to compare the IPs of different types of spoken stimuli (consonants, words, and final words in sentences) in both silence and noise conditions, using a gating paradigm (Grosjean, 1980). Subordinate to this aim were two more specific research questions. Firstly, *how much does noise generally affect IPs*? It was assumed that masking speech with noise would generally delay IPs. Secondly, *how does noise affect IPs when considering linguistic* (i.e., *lexical and sentential*) *context*? In consonant identification, compensatory lexical and contextual resources were not available in the present study. Therefore, listeners had to identify the consonants based on critical cues of their acoustic properties, distributed across their entire durations. In word identification, the masking of consonants and vowels with noise is likely to diminish one's ability to identify the words, or to misdirect the listener to interpret them as other words. However, lexical knowledge may aid listeners (Davis and Johnsrude, 2007), although noise is likely to delay IPs for words (as well as for consonants). In final word identification in sentences, we therefore assumed that the contextual and semantic information inherent in naturalistic sentences would speed up the identification of target words, even in noise, compared to words presented in isolation. Words positioned at the end of sentences that had either a low predictable or a high predictable semantic context were also compared, so as to further test the benefit of contextual support.

The second aim was to investigate the relationship between explicit cognitive functions (capacities of working memory and attention) and the IPs of different types of spoken stimuli (consonants, words, and final words in sentences) in both silence and noise conditions. On the basis of the ELU model (e.g., Rönnberg et al., 2008, 2013) as well as several independent empirical studies (e.g., Petkov et al., 2004; Snyder et al., 2006; Foo et al., 2007; Rudner et al., 2009, 2011), we predicted that significant correlations would exist between performance in tests of attention and working memory and IPs of gated stimuli in noise, but to a relatively lesser extent in silence.

### Methods

#### Participants

Twenty-one university students (12 males and 9 females) at Linköping University, Sweden were paid to participate in this study. Their ages ranged from 20 to 33 years (*M* = 24.6 years). All of the students were Swedish native speakers that spoke Swedish at home and at the university. According to the Swedish educational system, the students (or pupils) learn English and at least one another language (e.g., German, French, Spanish) in school. The participants reported having normal hearing, normal vision (or corrected-to-normal vision), and no psychological or neurological pathologies. The participants gave consent, pursuant to the ethical principles of the Swedish Research Council (Etikregler för humanistisk-samhällsvetenskaplig forskning, n.d.), the Regional Ethics Board in Linköping, and Swedish practice for research on normal populations.

### Measures

#### Gating speech tasks

***Consonants***. The study employed 18 Swedish consonants presented in vowel-consonant-vowel syllable format (/aba, ada, afa, aga, aja, aha, aka, ala, ama, ana, aŋa, apa, ara, aʈa, asa, aʃa, ata, ava/). The gate size for consonants was set at 16.67 ms. The gating started after the first vowel /a/ and right at the beginning of the consonant onset. Hence, the first gate included the vowel /a/ plus the initial 16.67^1^ ms of the consonant, the second gate gave an additional 16.67 ms of the consonant (a total of 33.34 ms of the consonant), and so on. The minimum, average, and maximum total duration of consonants were 85, 198, and 410 ms, respectively. The maximum number of gates required for identification was 25. The consonant gating task took between 40 and 50 min to complete.

***Words***. The words in this study were chosen from a pool of Swedish monosyllabic words in a consonant-vowel-consonant format that had average to high frequencies according to the Swedish language corpus PAROLE (2011). Forty-six of these words (all nouns) were chosen and divided into two lists (A and B) comprising 23 words each. Both lists were matched in terms of onset phonemes and neighborhood size (i.e., lexical candidates that shared similar features with the target word). Each word used in the present study had a small to average numbers of neighbors (3–6 alternative words with the same pronunciation of the first two phonemes, e.g., the target word /dop/ had the neighbors /dog, dok, don, dos/). For each participant, we presented one list in silence and the other in noise. The presentation of words was randomized across participants. Participants in the pilot studies complained that word identification with the gate size used for consonants (16.67 ms) led to fatigue and a loss of motivation. Therefore, a doubled gate size of 33.3 ms was used for word identification and also we presented the first phoneme (consonant) of each word as a whole, and gating was started from the onset of the second phoneme (vowel) in order to prevent any exhaustion for the participants. The minimum, average, and maximum duration of words were 548, 723, and 902 ms, respectively. The maximum number of gates required for identification was 21. The word gating task took between 35 and 40 min to complete.

***Final Words in Sentences***. There were two types of sentences in this study, which differed according to how predictable the last word in each sentence was: sentences with a highly predictable (HP) last word (e.g., “Lisa gick till biblioteket för att låna en *bok*”; “Lisa went to the library to borrow a *book*”) and sentences with a low predictable (LP) last word (e.g., “I förorten finns en fantastisk *dal*”; “In the suburb there is a fantastic *valley*”). The last (target) word in each sentence was always a monosyllabic noun.

To begin with, we constructed a battery of sentences that had differing predictability levels. This was followed by three consecutive pilot studies for the development of HP and LP sentences. First, the preliminary versions of sentences were presented in written form to some of the staff members at Linköping University in order to grade the predictability level of the target words in each sentence, from 0 (unpredictable) to 10 (highly predictable), and to obtain feedback on the content of the sentences in order to refine them. The sentences with scores over 7 were used as HP sentences, and those with scores below 3 were used as LP sentences. The rational for criterion below 3 for final words in LP sentences was based on our interest to have a minimum predictability in the sentences in order to separate identification of final words in LP sentences from identification of final words in anomalous sentences or identification of isolated-words. We then revised the sentences on the basis of the feedback. A second pilot study was conducted on 15 students at Linköping University to grade the predictability level of the revised sentences in the same way (from 0 to 10). Once again, the sentences with scores over 7 were used as HP sentences, and those with scores below 3 were used as LP sentences. In a third pilot study, the remaining sentences were presented to another 15 students to grade their predictability level. Again, we chose the sentences with scores over 7 as HP sentences, and the sentences with scores below 3 as LP sentences.

In total, there were 44 sentences (22 HP sentences and 22 LP sentences, based on the last word in each sentence). The gating started from the onset of the first phoneme of the target word. Because of the supportive effects of context on word identification, and based on the pilot data, we set the gate size at 16.67 ms to optimize time resolution. The average duration of each sentence was 3030 ms. The minimum, average, and maximum duration for target words at the end of sentences were 547, 710, and 896 ms, respectively. The maximum number of gates required for identification was 54. The gating final-word in sentence task took between 25 and 30 min to complete.

#### Hearing in Noise Test

We used a Swedish version of the HINT (Hällgren et al., 2006), adapted from Nilsson et al. (1994), to measure the hearing-in-noise ability of the participants. The HINT sentences consisted of three-to-seven word everyday sentences with fluctuating ±2 dB SNR. The sentences were normalized for naturalness, difficulty, and reliability. The sentences were read aloud by a female speaker. In the present study, we used one list consisting of 10 sentences in the practice test, and one list consisting of 20 sentences in the main test to estimate SNR required for 50% correct performance (i.e., correct repetition of 50% of the sentences). The HINT took about 10 min per participant to complete.

#### Cognitive tests

***Reading Span Test***. The reading span test was designed to measure working memory capacity. The task requires the retention and recall of words while reading simple sentences. Baddeley et al. (1985) developed one such test based on the technique devised by Daneman and Carpenter (1980) in which sentences are presented visually, word by word, on a computer screen.

Several small lists of short sentences were presented to participants on the screen. Each sentence had to be judged as to its semantic correctness. Half of the sentences were semantically correct, and the other half were not (e.g., “Pappan kramade dottern”; “The father hugged his daughter” or “Räven skrev poesi”; “The fox wrote poetry”) (Rönnberg et al., 1989; Rönnberg, 1990). The test began with two-sentence sets, followed by three-sentence sets, and so forth, up to five-sentence sets. Initially, participants were asked to press the “L” key if the sentence made sense or the “S” key for illogical sentences. After the set had been presented, participants were then required to recall either the first or final words of each sentence (e.g., “Pappan” and “Räven”; or “dottern”; and “poesi”), in the correct serial presentation order. Participants had about 3 s to press the “L” or “S” keys before the next sentence appeared. The computer instructed the participants to repeat either the first words or the last words of each sentence in the current set by typing them. The reading span score for each participant was equivalent to the total number of correctly recalled words across all sentences in the test, with a maximum score of 24. The reading span test took about 15 min per participant to complete.

***The Paced Auditory Serial Addition Test (PASAT)***. The PASAT was initially designed to estimate information processing speed (Gronwall, 1977), but it is widely considered a test of attention (for a review, see Tombaugh, 2006). The task requires subjects to listen to a series of numbers (1–9), and to add consecutive pairs of numbers as they listen. As each number is presented, subjects must add that number to the previous number. For example, the following sequence of numbers is presented, one number at a time, every 2 or 3 s: 2, 5, 7, 4, and 6. The answers are: 7, 12, 11, and 10. The test demands a high level of attention, particularly if the numbers are presented quickly. In this study, we used a version of the PASAT in which digits were presented at an interval of either 2 or 3 s (Rao et al., 1991), referred to as the PASAT 2 and the PASAT 3, respectively. Participants started with the PASAT 3, followed by the PASAT 2, with a short break between the two tests. The total number of correct responses (maximum possible = 60) at each pace was computed. The PASAT took about 15 min per participant to complete.

#### Preparation of gating tasks and procedure

A female speaker with clear enunciation and standard Swedish dialect read all of the items with normal intonation at a normal speaking rate in a quiet studio. Each item (consonant, word, or sentence) was recorded several times. We selected the item with the most natural intonation and clearest enunciation. Items were matched for sound level intensity. The sampling rate of the recording was 48 kHz, and the bit depth was 16 bits.

The onset and offset times of each recorded stimulus were marked in order to segment different types of stimuli. For each target, the onset time of the target was located as precisely as possible by inspecting the speech waveform (with Sound Studio 4 software) and using auditory feedback. The onset time was defined as the point where the signal amplitude ascended from the noise floor, according to the spectrograms in the Sound Studio 4 software. Each segmented section was then edited, verified, and saved as a “.wav” file. The gated stimuli were checked to eliminate click sounds. The root mean square value was computed for each stimulus waveform, and the stimuli were then rescaled to equate amplitude levels across the stimuli. A steady-state broadband noise, from Hällgren et al. (2006), was resampled and spectrally matched to the speech signals for use as background noise. The onset and offset of noise were simultaneous to the onset and offset of the speech signals.

The participants were tested individually in a quiet room. They were seated at a comfortable distance from a MacBook Pro (with Mac OS 10.6.7). Matlab (R2010b) was used to gate and present the stimuli binaurally through headphones (Sennheiser HDA200).

Participants received written instructions about the conditions for the different tasks (consonants, words, and final words in sentences), and performed several practice trials. In the practice trial, the sound level of the presentation was adjusted individually for each participant to a comfort level (approximately 60–65 dB). This sound level was used with no change in adjustment for that participant in both silent and noise conditions. In the noise condition (steady-state noise), the SNR was set at 0 dB, which was based on the findings of a pilot study using the current set of stimuli. During the practice session, the experimenter demonstrated how to use the keyboard to respond during the actual test. The participants were told that they would hear only part of a spoken target and would then hear progressively more. Participants were told to attempt identification after each presentation, regardless of how unsure they were about the identification of the stimulus, but to avoid random guessing. The participants were instructed to respond aloud and the experimenter recorded their responses. When necessary, the participants were asked to clarify their responses. The presentation of gates continued until the target was correctly identified on six consecutive presentations. If the target was not correctly identified, then the presentation continued until the entire target was disclosed, even if six or more consecutive responses were identical. Then, the experimenter started the next trial. When a target was not identified correctly, even after the whole target had been presented, its total duration plus one gate size was used as an estimate of the IP (cf. Elliott et al., 1987; Walley et al., 1995; Metsala, 1997; Hardison, 2005; Moradi et al., 2013). The rationale for this estimated IP was based on the fact that it was possible for participants to give correct responses at the last gate of a given target; hence, calculating an IP equal to the total duration of that target for two correct responses (even when late) and wrong responses would not be appropriate. No specific feedback was given to participants at any time during the session, except for general encouragement. Furthermore, there was no time pressure for responding to what was heard.

Each subject performed all of the gating tasks (consonants, words, and final words in sentences) in one session. All participants started with the identification of consonants task, followed by words task, and ended with the final words in sentences task. The type of condition (silence or noise) was counterbalanced across participants, such that half of the participants started with consonant identification in silence and then proceeded to consonant identification in noise, and vice versa for the other half of the participants. The order of items within each type of stimulus material (consonants, words, and sentences) varied between participants.

The full battery of gating tasks took 100–120 min per participant to complete. All of the tasks were performed in one session, but short rest periods were included to prevent fatigue. In the second session, the HINT, the reading span test, and the PASAT were administered. The order of the tests was counterbalanced across the participants. The second session took about 40 min per participant to complete.

## Results

### Gating speech tasks

**Figure 1:**
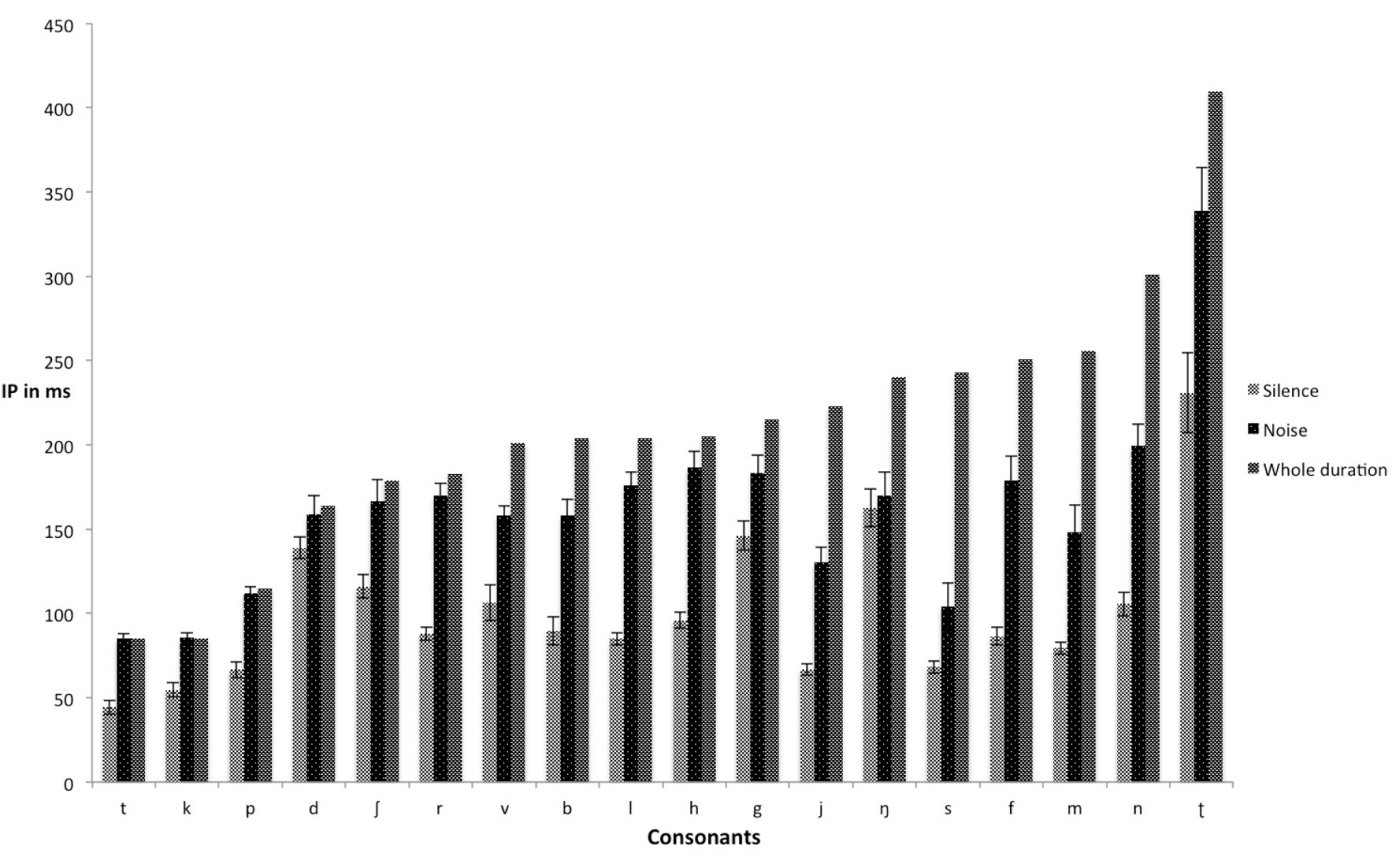
**Mean IPs (ms) for consonants in both silence and noise (with accompanying standard errors)**. IP, isolation point.

**Figure 2:**
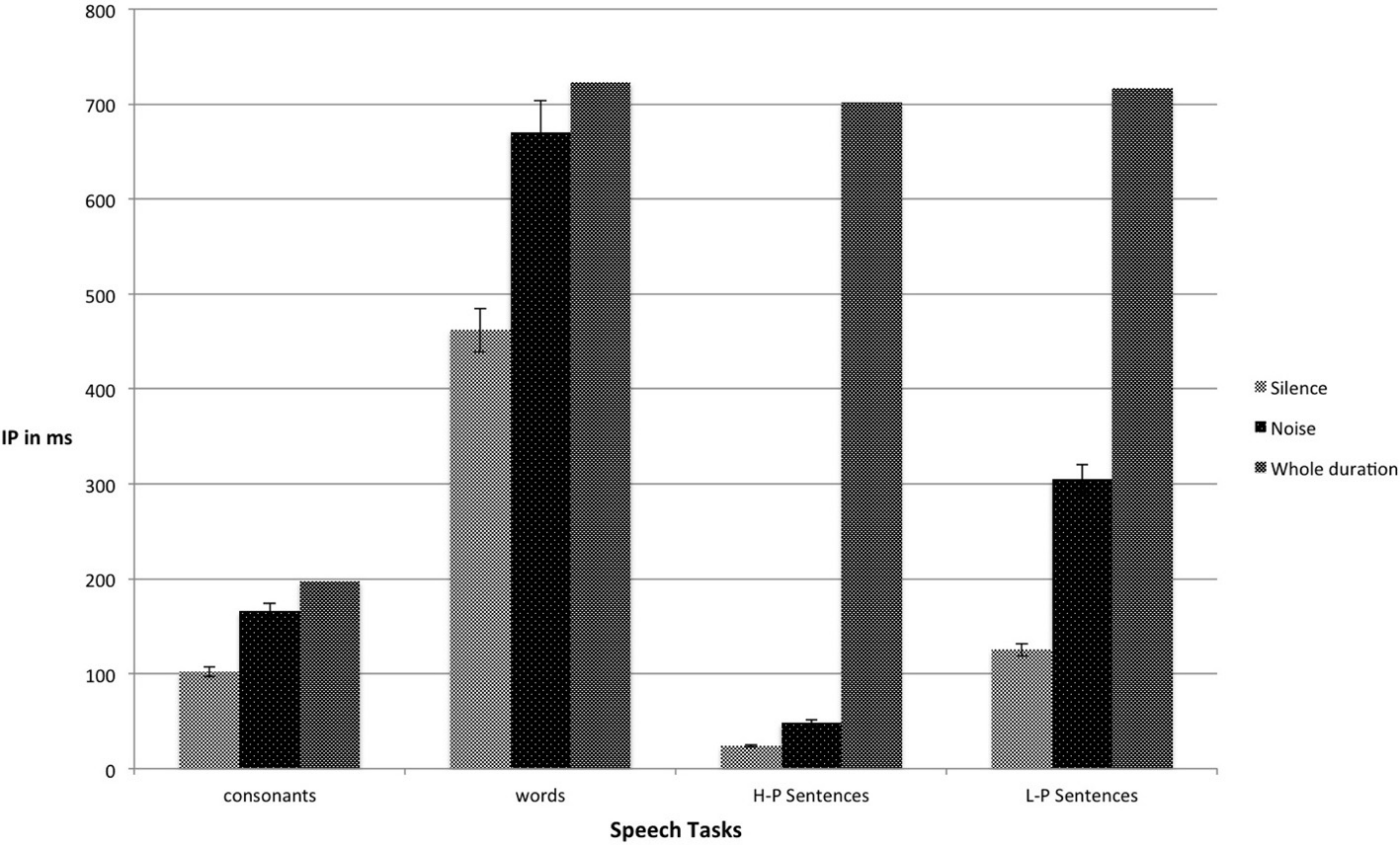
**Mean IPs (ms) for consonants, words, and final words in HP and LP sentences, in both silence and noise (with accompanying standard errors)**. IP, isolation point; HP, highly predictable; LP, low predictable.

Figure 1 shows the mean IPs of consonants presented in both silence and noise conditions. Appendices A and B are confusion matrices for the 18 Swedish consonants presented in silence and noise, respectively. The values in the confusion matrices were extracted from correct and incorrect responses across all gates in the consonant gating paradigm tasks performed either in silence and noise. Figure 2 shows the mean IPs for the gated speech tasks in both silence and noise conditions.

A Two-Way repeated-measure analysis of variance (ANOVA) was conducted to compare the mean IPs of the gated tasks (consonants, words, final words in LP sentences, and final words in HP sentences) in silence and noise. The results showed a main effect of the listening condition, *F*_(1, 20)_ = 213.54, *p* < 0.001, η*p*^2^ = 0.91; a main effect of the gated tasks, *F*_(1.23, 24.54)_ = 909.27, *p* < 0.001, η*p*^2^ = 0.98; and an interaction between listening condition and gated tasks, *F*_(1.58, 31.58)_ = 49.84, *p* < 0.001, η*p*^2^ = 0.71. Four planned comparisons showed that the mean IPs of *consonants* in silence (*M* = 101.78, *SD* = 11.47) occurred earlier than in noise (*M* = 166.14, *SD* = 26.57), *t*_(20)_ = 12.35, *p* < 0.001, *d* = 3.20. In addition, the mean IPs of *words* in silence (*M* = 461.97, *SD* = 28.08) occurred earlier than in noise (*M* = 670.51, *SD* = 37.64), *t*_(20)_ = 17.73, *p* < 0.001, *d* = 5.49. The mean IPs of *final words in LP sentences* in silence (*M* = 124.99, *SD* = 29.09) were earlier than in noise (*M* = 305.18, *SD* = 121.20), *t*_(20)_ = 7.67, *p* < 0.001, *d* = 2.56. In addition, the mean IPs of *final words in HP sentences* in silence (*M* = 23.96, *SD* = 3.31) occurred earlier than in noise (*M* = 48.57, *SD* = 23.01), *t*_(20)_ = 4.96, *p* < 0.001, *d* = 1.43. We also analyzed our data by including only correct responses. The results showed that the mean IPs for consonants were 98.26 (*SD* = 7.98) ms in silence and 137.83 (*SD* = 21.95) ms in noise. In words, the mean IPs in silence were 456.31 (*SD* = 21.49) ms in silence and 505.89 (*SD* = 50.77) ms in noise. In final words in LP sentences, the mean IPs were 102.18 (*SD* = 20.86) ms in silence and 114.94 (*SD* = 22.03) ms in noise. In final words in HP sentences, the mean IPs were 23.86 (*SD* = 3.33) ms in silence and 42.24 (*SD* = 15.24) ms in noise. When comparing the results from two methods of IP calculations (i.e., including error responses with whole IPs of target stimuli plus one gate size, vs. including correct responses only), there were subtle differences between IPs in silence; but greater differences in noise. For instance, when the IP calculation was based on correct responses only, the mean IPs for final word identification in sentences was 102.18 ms in silence and 114.94 ms in noise. However, when considering both correct and incorrect responses in the calculation of IPs for final word identification in sentences, the mean IPs became 124.99 ms in silence and 305.18 ms in noise. We therefore argue that the inclusion of error responses actually responses highlighted the interaction between noise and stimulus predictability (i.e., lexical, sentential, and semantic context), and that the interaction was logical and valid. In addition, the ANOVA on IPs only including correct responses showed the same pattern of results. There was a main effect of listening condition, *F*_(1, 20)_ = 45.89, *p* < 0.001, η*p*^2^ = 0.70; a main effect of the gated tasks, *F*_(1.68, 33.49)_ = 3545.27, *p* < 0.001, η*p*^2^ = 0.99; and an interaction between listening condition and gated tasks, *F*_(1.55, 30.91)_ = 6.10, *p* < 0.01, η*p*^2^ = 0.23.

Table 1 reports the percentage of correct responses for each of the gated tasks performed in both silence and noise conditions. A Two-Way repeated-measures analysis (ANOVA) showed a main effect of listening condition, *F*_(1, 20)_ = 223.41, *p* < 0.001, η*p*^2^ = 0.92; a main effect of the gated tasks, *F*_(3, 60)_ = 36.86, *p* < 0.001, η*p*^2^ = 0.65; and an interaction between listening condition and gated tasks, *F*_(3, 60)_ = 33.24, *p* < 0.001, η*p*^2^ = 0.62. Four planned comparisons showed that noise reduced the accuracy for the identification of consonants, *t*_(20)_ = 7.50, *p* < 0.001, *d* = 2.21; words, *t*_(20)_ = 15.14, *p* < 0.001, *d* = 4.26; final words in LP sentences, *t*_(20)_ = 4.28, *p* < 0.001, *d* = 1.10; and final words in HP sentences, *t*_(20)_ = 2.90, *p* < 0.009, *d* = 1.51.

**Table 1:** **Identification accuracy for gating spoken stimuli**.

**Type of gated stimuli**	**Silence mean (*SD*)**	**Noise mean (*SD*)**
Consonants	97.4 (3.8)	70.1 (17.5)
Words	96.3 (5.2)	34.6 (17.1)
HP Sentences	94.8 (7.7)	85.7 (8.0)
LP Sentences	87.3 (7.3)	67.1 (20.3)

SD, standard deviation; HP, highly predictable; LP, low predictable.

### Correlations between gating speech tasks, the HINT, and the cognitive tests

Table 2 shows the means responses of participants for the HINT, PASAT 3, PASAT 2, and the reading span test. The correlation matrix (Table 3) shows the Pearson correlations between the IPs of gated tasks in both silence and noise conditions (lower scores in the gated tasks reflect better function), the HINT scores (lower scores in the HINT reflect better function), and the reading span test and PASAT scores (higher scores in the reading span test and PASAT reflect better function). The PASAT 2 scores were significantly correlated with the HINT scores, the reading span test scores, IPs of consonants in noise, and IPs of words in noise. This finding suggested that lower IP scores for consonants and words in noise were usually associated with better performance on the HINT and PASAT 2. The reading span test scores were also significantly correlated with the HINT scores and IPs for consonants in noise, indicating that better performance on the reading span test was associated with better performance on the HINT and earlier IPs for consonants in noise. The HINT scores were significantly correlated with IPs for consonant and word identification in noise; the better the listeners performed on the HINT, the earlier they generally identified consonants and words in noise.

**Table 2:** **HINT, PASAT 3, PASAT 2, and reading span test results**.

**Type of task**	**Mean (SD)**
HINT	−3.1 (1.2)
PASAT 3	51.2 (4.4)
PASAT 2	40.0 (6.2)
Reading span test	21.6 (1.7)

HINT, Hearing in Noise Test; PASAT, Paced Auditory Serial Attention Test (digits are presented at an interval of 2 or 3 s); SD, standard deviation.

**Table 3:** **Correlation matrix for gating speech variables, HINT, and cognitive test results**.

	**1**	**2**	**3**	**4**	**5**	**6**	**7**	**8**	**9**	**10**	**11**	**12**
1. HINT		−0.09	−0.63^**^	−0.58^**^	0.27	0.73^**^	−0.26	0.58^**^	0.08	0.24	0.00	0.22
2. PASAT 3			0.51^*^	0.55^*^	−.012	−0.22	0.06	0.07	0.04	−0.14	−0.23	−0.39
3. PASAT 2				0.65^**^	−0.39	−0.68^**^	0.22	−0.51^*^	0.00	−0.21	0.03	−0.34
4. RST					−0.19	−0.51^*^	0.23	−0.30	−0.21	−0.41	−0.35	−0.42
5. Consonant-S						0.44^*^	−0.09	0.36	−0.15	0.03	0.07	0.32
6. Consonant-N							−0.03	0.56^**^	0.18	0.35	0.24	0.34
7. Word-S								−0.33	0.20	−0.11	−0.11	−0.27
8. Word-N									0.16	0.27	−0.16	0.16
9. HP-S										0.33	0.15	−0.04
10. LP-S											0.50^*^	0.56^**^
11. HP-N												0.58^**^
12. LP-N												

HINT, Hearing in Noise Test; PASAT, Paced Auditory Serial Attention Test (digits are presented at an interval of 2 or 3 s); RST, Reading Span Test; Consonant-S, gated consonant identification in silence; Consonant-N, gated consonant identification in noise; Word-S, gated word identification in silence; Word-N, gated word identification in noise; HP-S, gated final word identification in highly predictable sentences in silence; LP-S, gated final word identification in low predictable sentences in silence; HP-N, gated final word identification in high predictable sentences in noise; LP-N, gated final word identification in low predictable sentences in noise.

* *p < 0.05*.

** *p < 0.01*.

We also compared pairs of correlational coefficients in silence and noise (Table 4). The results showed that three pairwise correlations were significantly different from each other. We also tested if there is a difference between the means of the correlation coefficients of the two matrices (between the IPs and the scores of the cognitive tasks and the HINT, with *z* transformed correlation coefficients). We therefore first put all correlation coefficients in the same (logical) direction. Then we tested the means difference with a paired two-tailed *t* test. In this case, *n* = 12, since we used the number of paired correlations as “individuals.” The result was *t*_(10)_ = 3.64, *p* = 0.005, *d* = 1.05, that is, a significant difference between the mean correlation coefficients for silence versus noise, with a large effect size. We argue that the data pattern, comparing correlations for the silent versus noisy conditions, shows a valid difference such that cognitive tests are generally more strongly correlated with IPs for consonants and words in the noisy conditions compared to the silent conditions. Thus, support for the validity of this conclusion comes from (a) the overall qualitative pattern of differences in correlation matrices, (b) from inferential statistics comparing pairwise correlations, and (c) from statistical comparison of the entire (pooled) correlation matrices.

**Table 4:** **Fisher's Z scores to compare correlation coefficients between silence and noise**.

	**Consonants**	**Words**	**Final words in HP**	**Final words in LP**
HINT	−2.69^*^	−2.69^*^	0.26	0.09
PASAT 3	0.91	−0.03	1.02	1.23
PASAT 2	1.56	2.18^*^	−0.11	0.62
Reading span test	1.48	1.46	0.55	0.08

* *p < 0.05*.

## Discussion

### How does noise generally affect IPs?

The results show that noise generally delayed the IPs for the identification of consonants, words, and final words in LP and HP sentences, which is in line with the predictions. Furthermore, our results demonstrate the advantage of IPs over accuracy especially in the silent condition. While there was a ceiling effect for identification of consonants, words, and final words in HP sentences in silence (over 95% correct responses), there was substantial variation in their IPs.

### How does noise affect IPs when considering linguistic (i.e., lexical and sentential) context?

#### Consonants

There was variation in the IPs of consonants, implying that the location of critical cues for their identification varies across consonants, corroborating the findings of Smits (2000). For instance, the time ratio in silence showed that /b f h j l m n s/ required roughly one-third and /d k p ʃ/ required about two-thirds of their full durations for identification. Noise extended the amount of time required for correct identification of consonants. Consonants in the noise condition required longer exposure to be identified because their critical features were masked. In our study, the accuracy rate for correct identification of consonants was about 97% in silence, which dropped to 70% in noise (Table 1). This is consistent with the findings of Apoux and Healy (2011), wherein listeners correctly identified 68% of consonants in speech-shaped noise at 0 dB SNR. Cutler et al. (2008) reported about 98% correct identification of consonants in quiet conditions, and about 80% in eight-talker babble noise. In addition, the results in the confusion matrix (Supplementary meterials) for identification of Swedish consonants show that at 0 SNR dB, /b d g h k r ʈ ʃ t/ are often confused with each other, /f l m ŋ p r/ are moderately confused with each other, and /j n s/ hardly ever confused with each other.

#### Words

Noise also increased the amount of time required for the correct identification of Swedish monosyllabic words. In silence, just over half of the duration of a word was required for identification. This finding is consistent with previous studies using English words. Grosjean (1980) showed that about half of the segments of words were required for word identification. In noise, almost the full duration of words was required for identification in the current study. Table 3 shows that consonant identification in noise was significantly correlated with word identification in noise and HINT performance, which might imply that the misperception of a consonant was misleading for the identification of words in noise. In fact, the incorrect identification of just one consonant or vowel (in consonant-vowel-consonant word format) can lead to the activation of another candidate in the lexicon, and realizing the misperception and finding another candidate takes more time. In summary, noise delays word identification and increases the risk of misidentification, and may make it impossible to identify a word at all. This was also the case in the present study. Not only were the IPs delayed by noise, accuracy was also impeded: about 96% accuracy in silence versus 35% in noise (see Table 1). These results are also consistent with previous studies (Chermak and Dengerink, 1981; Studebaker et al., 1999).

#### Final words in sentences

The presence of noise delayed final word identification in LP and HP sentences. In silence, highly relevant contextual information seems to prohibit the activation of other lexical candidates even earlier than word-alone presentation. However, the presence of noise resulted in delayed identification of final words even in both LP and HP sentences. These results are in agreement with Aydelott and Bates (2004) who reported that the perceptual clarity of speech signal impacts on the ability to make use of semantic context to aid in lexical processing. They studied how response times to target words in congruent sentences were influenced by low-pass filtering of prior context. Their result showed that low-pass filtering reduced the facilitation of semantic context on identification of target words. The mean IPs for final-word identification in LP sentences (125 ms in silence and 305 ms in noise) were found to be even shorter than the mean IPs for isolated words in silence (462 ms), demonstrating that even low predictable information can speed up decoding of the speech signal (cf. Salasoo and Pisoni, 1985; Van Petten et al., 1999). The accuracy rates for final words in HP and LP sentences in noise were 86 and 67%, respectively, which also is consistent with Kalikow et al. (1977). As Table 1 shows, accuracy in the noise condition was higher for final words in LP sentences (67%) than for the identification of isolated words (35%). We assume that (similar to the identification of isolated words) masking consonants with noise activates other consonants which form words that are still related to the contents of LP sentences, and eliminating them is time consuming. However, because there is *some* contextual information in LP sentences that excludes *some* candidates in the mental lexicon, correct identification of final words in LP sentences is accomplished at earlier gates compared to the identification of words in isolation (cf. Ladefoged and Broadbent, 1957).

To conclude, the results from comparing IPs from gated speech stimuli in silence versus noise suggest that less information is available in noise because of masking (e.g., Dorman et al., 1998; Shannon et al., 2004; for a review, see Assmann and Summerfield, 2004). We suppose that the combination of noise with speech stimuli hindered the listener from accessing the detailed acoustic information (in particular for consonants and words), whereas this access to the detailed acoustic information was readily available in a silent condition. As a consequence, noise delays the amount of time required (in other words, necessitates more acoustic information) for correct identification of speech stimuli to occur. In addition, our finding is in agreement with the “active sensing” hypothesis (for a review see Zion Golumbic et al., 2012) which suggests that the brain consistently makes predictions about the identity of the forthcoming stimuli, rather than passively waiting to receive and thereafter identify the stimuli (Rönnberg et al., 2013).

### Cognitive demands of speech perception in silence and noise

#### HINT

Results showed that HINT performance was correlated with measures of working memory capacity (the reading span test), and attention capacity (PASAT 2). Listeners with better hearing-in-noise ability had higher scores in the tests of working memory and attention capacities. This result corroborates the previous studies that reported correlations between sentence comprehension in noise and the reading span test (e.g., Rudner et al., 2009; Ellis and Munro, 2013). Successful performance in the HINT requires filtering out the noise as well as focusing on the target signal, temporarily storing all of the words within sentences, and remembering them. It is therefore reasonable that HINT performance is correlated with the measures of attention and working memory capacities. One of the reasons for this correlation can be found in neuroimaging studies that demonstrate that the activation of auditory (superior temporal sulcus and superior temporal gyrus) *and* cognitive (e.g., left inferior frontal gyrus) brain areas are provoked during the comprehension of degraded sentences compared to clear speech (Davis et al., 2011; Wild et al., 2012; Zekveld et al., 2012). According to Giraud and Price (2001) and Indefrey and Cutler (2004), the tasks that require extra cognitive processes, such as attention and working memory, activate prefrontal brain areas that include the inferior frontal gyrus. Both stimulus degradation (Wild et al., 2012) and speech-in-noise seem to call on similar neurocognitive substrates (Zekveld et al., 2012). Thus, the observed HINT correlations are in agreement with previous studies.

#### Consonants

Better performance in the HINT, reading span test, and PASAT were associated with earlier identification of consonants in noise. Neuroimaging studies have also revealed that ambiguous phoneme identification requires top-down cognitive support from prefrontal brain areas in addition to predominantly auditory brain areas to correctly identify ambiguous phonemes (Dehaene-Lambertz et al., 2005; Dufor et al., 2007). However, our finding is not in agreement with Cervera et al. (2009) who showed no significant correlations between tests of working memory capacity (serial recall and digit ordering) and consonant identification in noise at 6 dB SNR. One explanation for this inconsistency may be the fact that we presented the gated consonants at 0 dB SNR, which is more difficult and cognitively demanding than the task used by Cervera et al. (2009).

#### Words in isolation

There was a significant correlation between the IPs of words in noise and scores for the HINT and PASAT 2, suggesting that listeners with better attention capacity and hearing-in-noise abilities identified words in noise earlier than those with poorer abilities. Shahin et al. (2009) degraded words by inserting white noise bursts around the affricatives and fricatives (of words). They found greater activation of the left inferior frontal gyrus during the processing of degraded words, which they suggested was implicated to “repair” the illusion of hearing words naturally when in reality participants had heard degraded words. In our study, it can be concluded that listeners who had better hearing-in-noise and attention capacities were able to repair this “illusion of hearing words naturally” earlier than those with poorer abilities, which resulted in shorter IPs for words in noise. It should be noted that we expected to see a significant correlation between IPs for words in noise and also with the reading span test (working memory capacity). However, there was no significant relationship between IPs for words in noise and test of working memory capacity. One explanation might be that for word identification, we presented the first phoneme of the words and then started the gating paradigm from the second phoneme (in a consonant-vowel-consonant format). In addition, the gate size for word identification was twice as large as for consonants. We therefore assume that this procedure for word identification reduced the demand on working memory for identification of words in noise. With the advantage of hindsight, this potentially important procedural detail should be accounted for in future gating research.

Overall, our findings for the identification of consonants and words in silence and noise are consistent with general predictions of the ELU model (Rönnberg et al., 2008, 2013), which suggests that speech perception is mostly effortless under optimum listening conditions, but becomes effortful (cognitively demanding) in degraded listening conditions. Clearly audible signals may not depend as much on working memory and attentional capacities, because they can be implicitly and automatically mapped onto the phonological representations in the mental lexicon.

#### Final words in sentences

Our results showed that there were no correlations between the IPs for final words in HP and LP sentences in noise condition and measures of working memory and attention. This finding is consistent with some previous studies which have shown that when listening is challenged by noise, prior contextual knowledge acts as a major source of disambiguation by providing expectations about which word (or words) may appear at the end of a given sentence (cf. Cordillo et al., 2004; Obleser et al., 2007). Hence, it can be assumed that at an equal SNR, the identification of final words in sentences is easier than the identification of consonants and words uttered in isolation; the sentence context makes final word identification less cognitively demanding (i.e., less effortful) than the identification of isolated consonants and words. This result is not in agreement with the original version of the ELU model (Rönnberg, 2003; Rönnberg et al., 2008) in which there was no postulated mechanism for the contextual elimination of lexical candidates. However, in the recent updated version of the ELU model (Rönnberg et al., 2013), the early top-down influence of semantic context on speech recognition under adverse conditions is taken into account. The model suggests that because of the combined semantic and syntactic constraints in a given dialog, listeners may need little information regarding a target signal, if the preceding contextual priming is sufficiently predictive.

In our study, while there were correlations between measures of cognitive tests and the HINT, no significant correlations were observed between cognitive tests and the IPs of final words in (LP and HP) sentences. One possible explanation might be that performance on the HINT requires listeners to remember *all* of the words in each sentence correctly, at varying SNRs, which taxes working memory (Rudner et al., 2009; Ellis and Munro, 2013). Successful performance in this task requires the short-term decoding and maintenance of masked speech stimuli, and the subsequent retrieval of the whole sentence. However, the identification of final words in sentences simply requires the tracking of incoming speech stimuli, and the subsequent guessing of the final words is based on the sentential context and the first consonant of the final word. This prior context plus initial consonant is likely to reduce cognitive demands, which was presumably lower than that required for the HINT performance. In addition, performance in the HINT was based on 50% correct comprehension of sentences in noise. As Table 1 shows, the mean accuracy rates in the noise condition for final words in LP and HP sentences were about 67 and 86%, respectively, which are higher than the 50% correct comprehension rate for sentences in the HINT. Furthermore, the mean SNR for HINT performance in the present study was −3.1 dB (Table 2), while final words in sentences in noise condition were presented at 0 dB. Thus, it can be concluded that identification in the LP and HP sentences under the noise condition was easier than HINT identification, and as such tapped into the implicit mode of processing postulated by the ELU model. Future studies are needed in order to investigate the correlations between tests of working memory and attention and IPs for final-word identification in sentences at lower SNRs. It is likely that by decreasing the SNR, the demand on working memory and attention capacities will increase even for such sentence completion tasks.

In our study, the PASAT demonstrated a significant correlation with the reading span test, which is in agreement with previous studies (Sherman et al., 1997; Shucard et al., 2004). Interestingly, only the PASAT 2 was correlated with HINT performance and consonant and word identification in noise, whereas the PASAT 3 was not. This probably suggests that the significant relationship with speech perception in noise was related to the attention-demanding aspect of the task, because PASAT 2 is more paced and taxing. This result is in line with the review by Akeroyd (2008), who argued that only sufficiently taxing cognitive tasks are correlated with speech perception in degraded listening conditions. In Akeroyd (2008), not all cognitive tests yielded significant correlations with noise; only specific measures of cognitive abilities such as working memory (e.g., the reading span test) were correlated with speech-in-noise tasks, whereas general, composite, cognitive measures (like IQ) were not.

Taken together, noise delays the IPs for identification of speech stimuli. In addition, the results suggest that early and correct identification of spoken signals in noise requires an interaction between auditory, cognitive, and linguistic factors. Speech tasks that lack a contextual cue, such as consonants and words presented in isolation, more probably draw on the interaction between auditory and explicit cognitive factors. However, when the perception of speech in noise relies on prior contextual information, or when there is no noise, superior auditory and cognitive abilities are less critical.

## Conclusions

The identification of consonants, words, and final words in sentences was delayed by noise. The mean correlation between cognitive tests and IPs was stronger for the noisy condition than for the silent condition. Better performance in the HINT was correlated with greater capacities of working memory and attention. Rapid identification of consonants in noise was associated with greater capacities of working memory and attention and also HINT performance; and rapid identification of words in noise was associated with greater capacity of attention and HINT performance. However, the identification of final words in sentences in the noise condition was not demanding enough to depend on working memory and attentional capacities to aid identification. This is presumably due to the facilitation from prior sentential context, lowering the demands on explicit cognitive resources.

### Conflict of interest statement

The authors declare that the research was conducted in the absence of any commercial or financial relationships that could be construed as a potential conflict of interest.

## Footnotes

^1^The rationale for setting gate size to 16.67 ms came from audiovisual gating tasks (See Moradi et al., 2013), to get the same gate size for both conditions (i.e., audiovisual and auditory modalities). By using 120 frames/s for recording visual speech stimuli, 8.33 ms of a visual stimulus is available in each frame (1000 ms/120 frame/s = 8.33 ms). Multiplying 8.33 by 2 (frames), there is 16.67 ms (Please see Lidestam, 2014, for detailed information).
