# Supplementary material for: Gated auditory speech perception: effects of listening conditions and cognitive capacity
Source: Front Psychol. 2014 Jun 2;5:531. doi: 10.3389/fpsyg.2014.00531 (PMC4040882; doi:10.3389/fpsyg.2014.00531)
Supplement: Supplementary file 1 [file DataSheet1.DOCX]

Appendix A. *Confusion matrix for 18 Swedish consonants presented in silence**

|  | b | d | f | g | h | j | k | l | m | n | ŋ | p | r | ʈ | s | ʃ | t | v |
| --- | --- | --- | --- | --- | --- | --- | --- | --- | --- | --- | --- | --- | --- | --- | --- | --- | --- | --- |
| b | 21 |  |  |  |  |  |  |  |  |  |  |  |  |  |  |  |  |  |
| d |  | 20 |  |  |  |  |  |  |  |  |  |  |  |  |  |  | 1 |  |
| f |  |  | 21 |  |  |  |  |  |  |  |  |  |  |  |  |  |  |  |
| g |  |  |  | 20 |  |  |  |  |  |  | 1 |  |  |  |  |  |  |  |
| h |  |  |  |  | 21 |  |  |  |  |  |  |  |  |  |  |  |  |  |
| j |  |  |  |  |  | 21 |  |  |  |  |  |  |  |  |  |  |  |  |
| k |  |  |  |  |  |  | 21 |  |  |  |  |  |  |  |  |  |  |  |
| l |  |  |  |  |  |  |  | 21 |  |  |  |  |  |  |  |  |  |  |
| m |  |  |  |  |  |  |  |  | 21 |  |  |  |  |  |  |  |  |  |
| n |  |  |  |  |  |  |  |  |  | 21 |  |  |  |  |  |  |  |  |
| ŋ |  |  |  |  |  |  |  |  |  | 2 | 19 |  |  |  |  |  |  |  |
| p |  |  |  |  |  |  |  |  |  |  |  | 21 |  |  |  |  |  |  |
| r |  |  |  |  |  |  |  |  |  |  |  |  | 21 |  |  |  |  |  |
| ʈ |  |  |  |  |  |  |  |  |  |  |  |  |  | 17 |  |  | 4 |  |
| s |  |  |  |  |  |  |  |  |  |  |  |  |  |  | 21 |  |  |  |
| ʃ |  |  |  |  |  |  |  |  |  |  |  |  |  |  |  | 21 |  |  |
| t |  |  |  |  |  |  |  |  |  |  |  |  |  |  |  |  | 21 |  |
| v |  |  | 1 |  |  |  |  |  |  |  |  |  |  |  |  |  |  | 20 |

*Data extracted from correct and incorrect responses across all gates. Rows represent the consonants presented, and columns represent participants’ responses.

Appendix B. *Confusion matrix for 18 Swedish consonants presented in noise**

|  | b | d | f | g | h | j | k | l | m | n | ŋ | p | r | ʈ | s | ʃ | t | v |
| --- | --- | --- | --- | --- | --- | --- | --- | --- | --- | --- | --- | --- | --- | --- | --- | --- | --- | --- |
| b | 14 | 1 | 1 |  |  |  |  |  | 1 |  |  | 1 |  |  |  |  |  | 2 |
| d | 3 | 14 |  |  |  | 1 |  |  |  |  |  |  |  |  |  |  | 1 | 3 |
| f |  |  | 16 |  |  |  |  |  |  |  |  |  |  |  | 3 | 1 |  | 1 |
| g |  |  |  | 13 |  | 4 | 1 |  |  |  | 3 |  |  |  |  |  |  |  |
| h | 1 | 1 |  |  | 11 |  | 2 | 2 | 1 |  |  |  |  |  | 1 | 2 |  |  |
| j |  |  |  |  |  | 20 |  |  |  |  | 1 |  |  |  |  |  |  |  |
| k |  |  |  |  | 1 |  | 13 |  |  |  |  |  |  | 2 |  |  | 5 |  |
| l |  | 1 |  |  |  | 1 |  | 16 |  | 2 |  |  | 1 |  |  |  |  |  |
| m |  |  |  |  |  |  |  |  | 17 | 1 | 2 |  |  |  |  |  |  | 1 |
| n |  |  |  |  |  |  |  |  |  | 19 | 1 |  |  |  |  |  |  | 1 |
| ŋ |  |  |  | 2 |  | 4 |  |  |  |  | 15 |  |  |  |  |  |  |  |
| p |  | 1 | 1 |  |  |  | 3 |  |  |  |  | 16 |  |  |  |  |  |  |
| r | 1 | 2 |  | 2 |  |  |  | 2 |  | 1 |  |  | 11 |  |  |  |  | 2 |
| ʈ |  | 1 |  |  | 2 |  |  |  |  |  |  | 1 |  | 9 | 1 | 2 | 5 |  |
| s |  |  | 1 |  |  |  |  |  |  |  |  |  |  |  | 19 | 1 |  |  |
| ʃ |  |  | 2 |  |  | 1 | 2 |  |  |  |  | 1 |  | 1 |  | 13 |  | 1 |
| t |  | 1 |  |  |  |  | 4 |  |  |  |  | 1 |  | 2 |  |  | 13 |  |
| v | 3 |  |  |  |  |  |  |  | 1 |  |  |  |  |  | 1 |  |  | 16 |

*Data extracted from correct and incorrect responses across all gates. Rows represent the consonants presented, and columns represent participants’ responses.
